# Detection of *Leishmania tarentolae* in lizards, sand flies and dogs in southern Italy, where *Leishmania infantum* is endemic: hindrances and opportunities

**DOI:** 10.1186/s13071-021-04973-2

**Published:** 2021-09-08

**Authors:** Jairo Alfonso Mendoza-Roldan, Maria Stefania Latrofa, Roberta Iatta, Ranju R. S. Manoj, Rossella Panarese, Giada Annoscia, Marco Pombi, Andrea Zatelli, Fred Beugnet, Domenico Otranto

**Affiliations:** 1 Dipartimento di Medicina Veterinaria, Università degli studi Di Bari, 70010 Valenzano, Italy; 2 Dipartimento di Sanità Pubblica E Malattie Infettive, “Sapienza” Università di Roma - Piazzale Aldo Moro 5, 00185 Rome, Italy; 3 Boehringer-Ingelheim, Avenue Tony Garnier, 29, 69007 Lyon, France; 4 Faculty of Veterinary Sciences, Bu-Ali Sina University, Hamedan, Iran

**Keywords:** Canine leishmaniasis, dqPCR, IFAT, *Leishmania infantum*, *Leishmania tarentolae*, Reptiles, *Sergentomyia minuta*, Zoonosis

## Abstract

**Background:**

*Leishmania tarentolae* is a protozoan isolated from geckoes (*Tarentola annularis*, *Tarentola mauritanica*), which is considered non-pathogenic and is transmitted by herpetophilic *Sergentomyia* spp. sand flies. This species occurs in sympatry with *Leishmania infantum* in areas where canine leishmaniasis is endemic. In the present study, we investigated the circulation of *L. tarentolae* and *L. infantum* in sand flies, dogs and lizards in a dog shelter in southern Italy, where canine leishmaniasis by *L. infantum* is endemic.

**Methods:**

Sheltered dogs (*n* = 100) negative for *Leishmania* spp. (March 2020) were screened by immunofluorescence antibody test (IFAT) using promastigotes of both species at two time points (June 2020 and March 2021). Whole blood from dogs, tissues of *Podarcis siculus* lizards (*n* = 28) and sand flies (*n* = 2306) were also sampled and tested by a duplex real-time PCR (dqPCR). Host blood meal was assessed in sand flies by PCR.

**Results:**

Overall, 16 dogs became positive for *L. infantum* and/or *L. tarentolae* by IFAT at one or both sampling periods. One canine blood sample was positive for *L. infantum*, whilst two for *L. tarentolae* by dqPCR. At the cytology of lizard blood, *Leishmania* spp. amastigote-like forms were detected in erythrocytes. Twenty-two tissue samples, mostly lung (21.4%), scored molecularly positive for *L. tarentolae*, corresponding to 10 lizards (i.e., 35.7%). Of the female *Sergentomyia minuta* sampled (*n* = 1252), 158 scored positive for *L. tarentolae*, four for *L. infantum*, and one co-infected. Two *Phlebotomus perniciosus* (out of 29 females) were positive for *L. tarentolae*. Engorged *S. minuta* (*n* = 10) fed on humans, and one *P. perniciosus*, positive for *L. tarentolae*, on lagomorphs.

**Conclusions:**

Dogs and lacertid lizards (*Podarcis siculus*) were herein found for the first time infected by *L. tarentolae*. The detection of both *L. tarentolae* and *L. infantum* in *S. minuta* and *P. perniciosus* suggests their sympatric circulation, with a potential overlap in vertebrate hosts. The interactions between *L. tarentolae* and *L. infantum* should be further investigated in both vectors and vertebrate hosts to understand the potential implications for the diagnosis and control of canine leishmaniasis in endemic areas.

**Graphical abstract:**

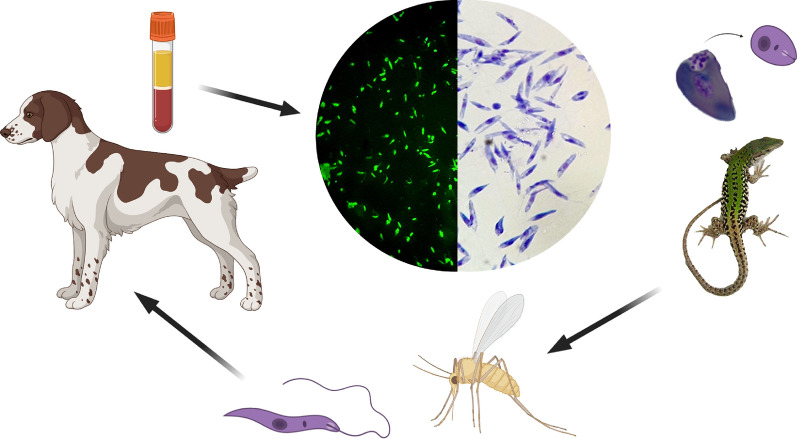

## Background

Zoonotic visceral leishmaniasis, caused by *Leishmania infantum* (Kinetoplastida, Trypanosomatidae), is a neglected disease of medical and veterinary importance, which impacts health, society and the economy in many tropical, subtropical and temperate regions of the globe [1]. Indeed, this disease affects mainly poor people [2] and may be fatal if not treated timely and properly. Infected dogs are the main reservoirs of *L. infantum* in the domestic and peri-domestic environments [3], with mainly subclinical presentation and only a small proportion manifesting overt clinical disease [4]. The causative agent is transmitted by bites of phlebotomine sand flies of the genera *Phlebotomus* in the Old World [5, 6] and *Lutzomyia* in the New World [7, 8]. Meanwhile, phlebotomine sand flies of the genus *Sergentomyia* are known to feed primarily on cold-blooded animals [9] and are associated to *Leishmania* spp. in lizards [9–12]. Nonetheless, DNA of *L. infantum* has been detected in *Sergentomyia minuta* [13, 14], suggesting it can feed also on available endothermic tetrapod animals. This picture has also been corroborated by other reports of *L. infantum* DNA in several *Sergentomyia* spp.*,* such as *Sergentomyia dubia*, *Sergentomyia magna* and *Sergentomyia schewtzi* in Africa [15], and *S. minuta* from endemic areas of canine leishmaniasis (CanL) in Europe [13, 14, 16–21]. Meanwhile, the DNA and/or amastigote forms of pathogenic *Leishmania* spp. (i.e., *Leishmania donovani*, *Leishmania tropica* and *Leishmania turanica*) have been detected in reptiles [22–25], therefore suggesting their potential role as reservoirs of mammalian pathogenic *Leishmania* spp. [24, 26, 27]. On the other hand, *Leishmania tarentolae* (subgenus *Sauroleishmania*) is a less regarded trypanosomatid infecting geckoes (e.g., *Tarentola mauritanica*), possibly transmitted by herpetophilic *Sergentomyia* spp. in Europe, North Africa and the Middle East [28, 29]. Incidentally, *L. tarentolae* is widely considered non-pathogenic. Nonetheless, some strains of this species (e.g., RTAR/FR/78/LEM125) may cause transient infection in mammals under laboratory conditions, as this species can differentiate into amastigote-like forms [30–33]. However, the molecular findings of *L. tarentolae* in a human mummy in Brazil [33], as well as in human blood [14] in central Italy, suggest its capacity to infect mammals. Nevertheless, the pathogenicity, virulence and overall deleterious effects of *L. tarentolae* in mammals are still unknown. In addition, given the high similarity in gene composition with *L. infantum* (i.e., 90%), *L. tarentolae* is considered a model for recombinant protein production and vaccine candidate [34–36], which could mean that natural infection with *L. tarentolae* may have a protective effect against *L. infantum* [37]. Given the variations in dog antibody levels between seasons of sand fly activity and the sympatric occurrence of both *Leishmania* species, we investigated the circulation of *L. tarentolae* and *L. infantum* in sand flies, dogs and lizards in a dog shelter in southern Italy where CanL by *L. infantum* is endemic.

## Methods

### Study area and sample collection

**Fig. 1:**
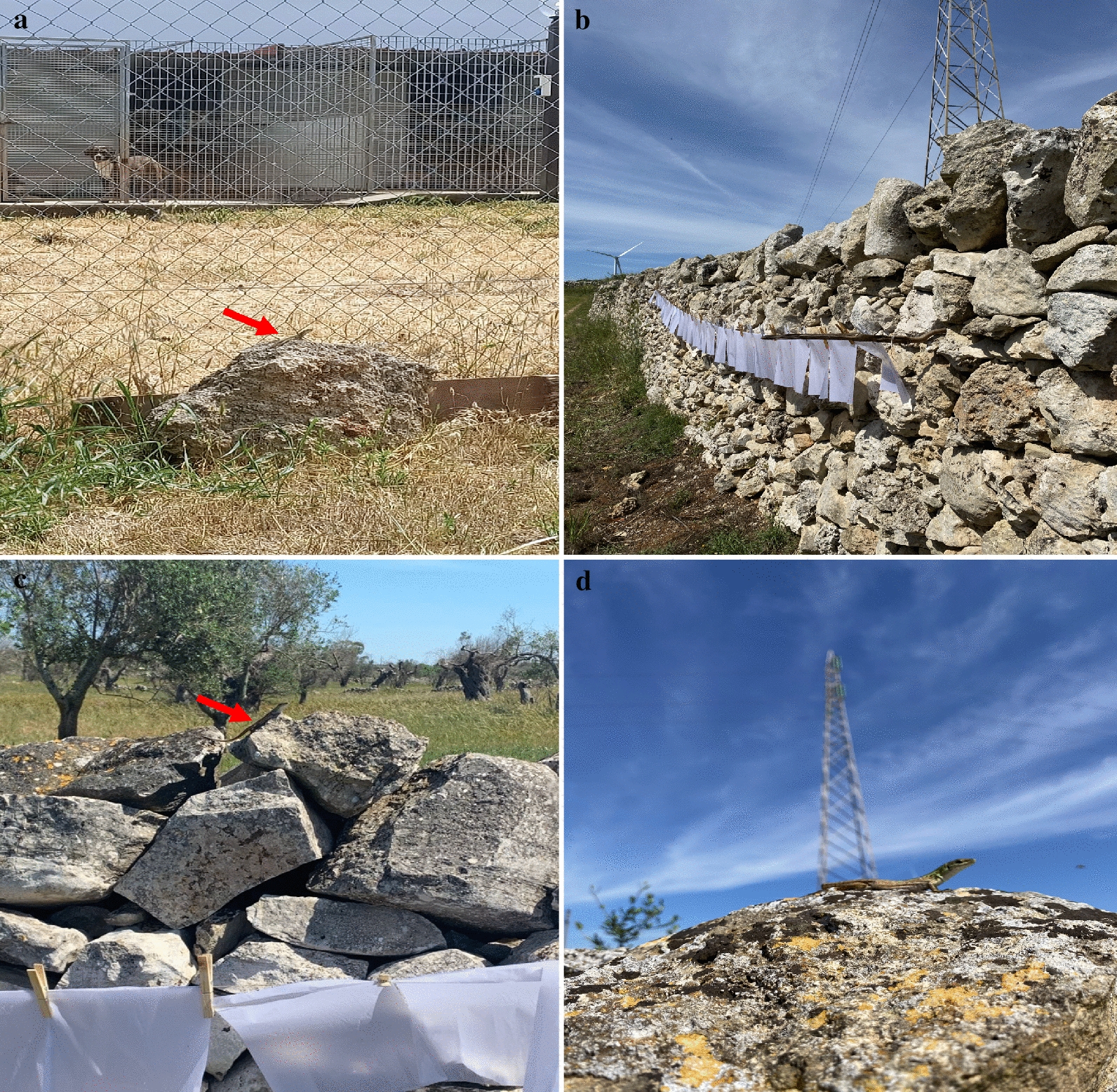
Dog shelter characteristics, and sand fly and lizard capture in a canine leishmaniasis-endemic area. **a** Lizard near the dog households (red arrow). **b** Sticky trap placement on the surrounding *muretti a secco*. **c** Lizard near the sticky traps (red arrow). **d** *Podarcis siculus* lizard

One hundred dogs which scored negative to *Leishmania* spp. on molecular and serological tests in March 2020 were re-sampled in June 2020 and March 2021 in a shelter located in a CanL-endemic area in Apulia region, southern Italy (40.419326N, 18.165582E, Lecce) [38]. The shelter is built in a dry and windy area 8.0 km from the nearest seaside (Fig. 1a). The environment around the shelter is characterized by few olive trees, withered grass, no water sources and surrounded by the typical *muretti a secco* (stone walls) where reptiles (i.e., *Podarcis siculus* lizards, *Hierophis viridiflavus carbonarius* snakes, and *T. mauritanica* geckoes) and rodents thrive. Dogs with a minimum age of 7 months were included in the study; signalment (i.e., age, sex, breed) and anamnestic data (i.e., previous protozoan and bacterial infection, and treatment) were recorded at time of enrollment. Moreover, a complete physical examination was performed by a veterinary clinician to assess the health status of the enrolled dogs. From each dog, whole blood was collected in vacuum containers with EDTA (2.5 ml) and serum collection tubes with clot activator (5 ml).

From May to November 2020, sand flies were collected biweekly using 64 sticky papers (21.0 cm × 29.7 cm, covering up to 4 m^2^) (Fig. 1b) and two CDC light traps were set from 5:00 p.m. to 8:00 a.m. Collections were carried out during the sand fly activity season [16] until the total disappearance/absence of sand flies (i.e., three consecutive negative captures). All specimens were stored in labeled glass vials containing 70% ethanol then morphologically identified using taxonomic keys and descriptions [39, 40].

Reptiles were captured in the area of the shelter, on the same walls where the sticky traps were placed (Fig. 1c), by lassoing or by hand. Species of reptiles were identified using reference keys [41], and then physically examined to assess their health status. Anamnestic data (e.g., species, biological stage, sex, physical abnormalities such as tail loss or predator-induced wounds) were recorded in each animal’s file. A small amount of blood was obtained via lizard tail fracture or by cardiocentesis when animals were adults and non-gravid females. Blood samples were stored at −20 °C and tail tissue in 70% ethanol. For each animal, blood smears were performed and then assessed for the presence of *Leishmania* parasites [42] using Diff-Quik stain [43]. Smears were rinsed in tap water to remove excess stain, and later evaluated using an optical microscope (LEICA DM LB2, Germany). Fecal samples were also collected from each animal. Captured lizards were humanely euthanized according to protocols [44] and dissected. Intestine, heart, kidneys, liver, lungs, spleen and skeletal muscle were individually collected and frozen at −20 °C.

### Serological testing

**Fig. 2:**
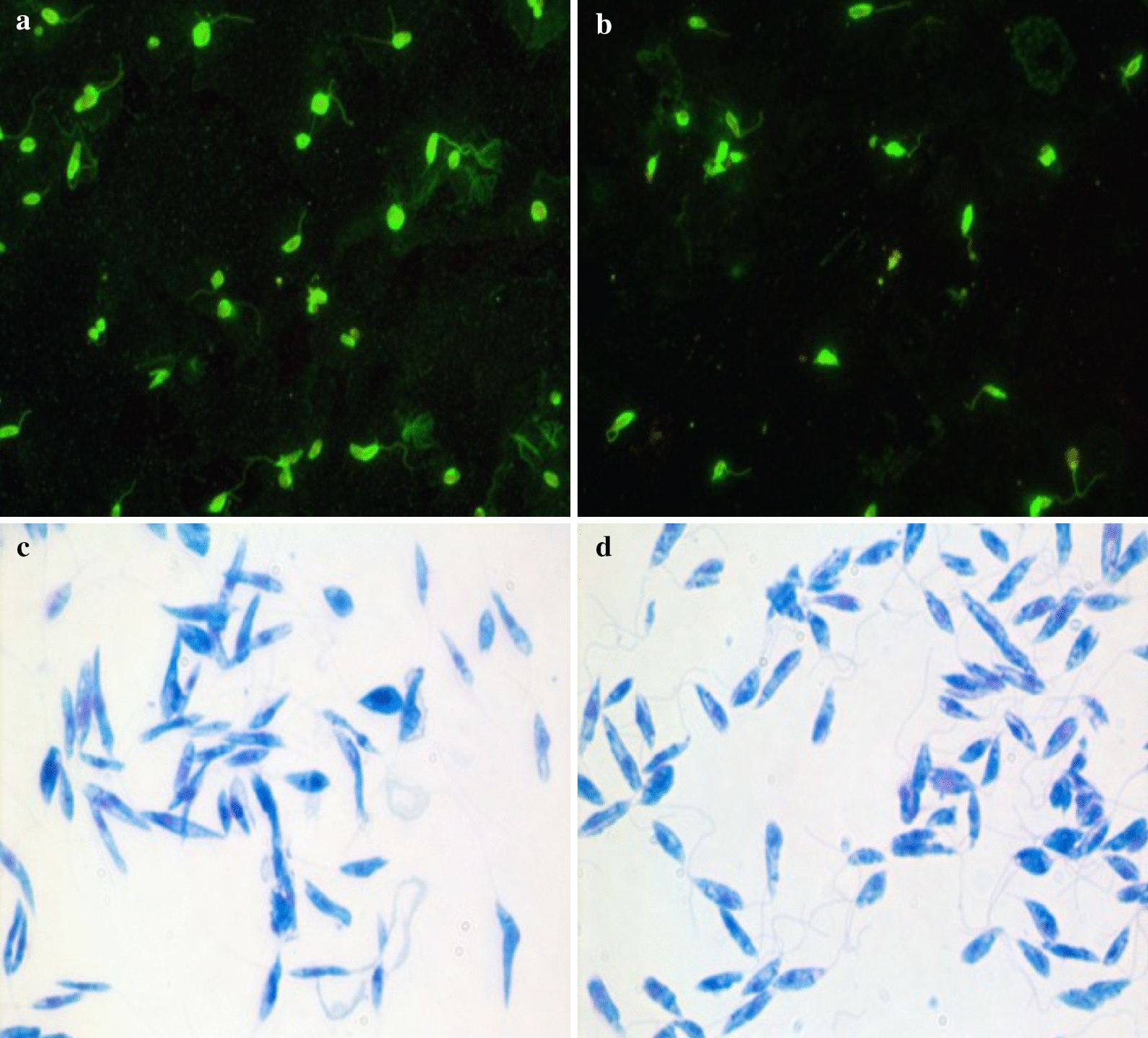
IFAT and cultured promastigotes of *Leishmania infantum* and *Leishmania tarentolae*. **a** IFAT using as antigen promastigotes of *L. infantum* (zymodeme MON-1). **b** IFAT using as antigen promastigotes of *L. tarentolae* (strain RTAR/IT/81/ISS21-G.6c). **c** Cultured promastigotes of *L. infantum* (zymodeme MON-1). **d** Promastigotes of *L. tarentolae* (strain RTAR/IT/81/ISS21-G.6c)

Serum samples from all enrolled dogs were tested to assess the exposure to *L. infantum* and *L. tarentolae*. An IFAT for the detection of IgG anti-*L. infantum* was performed as previously described (Fig. 2a) [45], whereas for antibodies against *L. tarentolae*, the IFAT was performed using promastigotes of *L. tarentolae* (strain RTAR/IT/81/ISS21-G.6c) as antigen (Fig. 2b) following the same procedure as for *L. infantum* IFAT. Serum samples from a dog positive for *L. infantum* by cytological and molecular analyses, and a healthy dog negative for *L. infantum*, were used as positive and negative controls, respectively, for both IFAT. Samples were scored as positive when they produced a clear cytoplasmic and membrane fluorescence of promastigotes from a cut-off dilution of 1:80 [46]. Positive sera were titrated by serial dilutions until negative results were obtained.

### Molecular procedures

Genomic DNA (gDNA) was extracted from the thorax and abdomen (heads and last segments were removed for morphological identification) of each female sand fly (*n* = 1281) using an in-house method as previously described [47]. Lizard tissues (i.e., intestine, heart, kidneys, liver, lungs, spleen, skeletal muscle and tail), as well as blood samples from lizards and dogs, were extracted using two commercial kits, GenUP gDNA and GenUP Blood DNA kits (Biotechrabbit GmbH, Hennigsdorf, Germany), respectively, according to the manufacturer’s instructions. DNA from lizard fecal samples was extracted using a specific commercial kit (DNeasy PowerSoil Kit, QIAGEN, Hilden Germany) following the manufacturer’s instructions. All samples were tested by duplex real-time PCR (dqPCR) for detection of *L. infantum* and *L. tarentolae* (samples were considered positive with quantitation cycle (Cq) values up to 38.0 and 38.6, respectively), as previously described [48]. Approximately 100 ng of gDNA (with the exception of the no-template control) was added to each dqPCR. gDNA from cultured promastigotes of *L. infantum*, originally retrieved from a dog living in Italy (zymodeme MON-1) (Fig. 2c), and *L. tarentolae* (strain RTAR/IT/81/ISS21-G.6c) (Fig. 2d) was used as positive controls. For sequences analyses, *Leishmania* dqPCR-positive samples were amplified by conventional PCR (cPCR) using primers L5.8S/LITSR targeting the partial region of the internal transcribed spacer 1 (ITS1, ~ 300 bp) and PCR protocol run as described elsewhere [49].

Engorged sand flies (*n* = 22) and all specimens that scored positive for *Leishmania* spp. were tested for blood-meal determination by cPCR using primers targeting the vertebrate host mitochondrial cytochrome *b* (350 bp), and a PCR protocol was run as previously described [19]. All PCR reactions consisted of 4 μl of gDNA and 46 μl of PCR mix containing 3 mM MgCl2, 10 mM Tris–HCl (pH 8.3) and 50 mM KCl, 125 μM of each dNTP, 1 pmol/μl of each primer and 2 U of AmpliTaq Gold (Applied Biosystems, Foster City, CA, USA). Amplified products were examined on 2% agarose gels stained with GelRed (VWR International PBI, Milan, Italy) and visualized on a Gel Logic 100 gel documentation system (Kodak, NY, USA). Amplicons were purified and sequenced in both directions using the same primers as for PCR, employing the Big Dye Terminator v.3.1 chemistry in an automated sequencer (3130 Genetic Analyzer, Applied Biosystems, Foster City, CA, USA). All sequences were aligned using the ClustalW program [50] and compared with those available in GenBank using the BLASTn tool (http://blast.ncbi.nlm.nih.gov/Blast.cgi).

To determine genetic clustering of *L. tarentolae*, the representative ITS1 sequences obtained from lizard, sand fly and dog samples and from reference strains of *L. tarentolae* and *L. infantum* were phylogenetically analyzed along with those of other *Leishmania* spp. available in the GenBank database. Phylogenetic relationships were inferred using the maximum likelihood (ML) method based on the Kimura 2-parameter model [51], and discrete gamma distribution was used to model evolutionary rate differences among sites, selected by best-fit model analysis and based on the lowest score obtained by Bayesian information criterion (BCI) using MEGA6 software [51]. Evolutionary analyses were conducted with 5000 bootstrap replications using MEGA6 software [52]. The corresponding ITS1 sequence of *Trypanosoma brucei* (GenBank: KU552356.1) was used as outgroup.

## Results

Of 100 dogs serologically examined, 16 scored positive against promastigotes of *L. infantum* and/or *L. tarentolae* by IFAT at one or both sampling periods (June 2020 and March 2021; Table 1). In particular, three dogs scored positive only against promastigotes of *L. infantum* (titer of 1:80) and five of *L. tarentolae* (titer up to 1:160). Of the eight animals positive for both species, four were positive at both time points, the remaining with different combinations (Table 1). Of dog blood samples tested by dqPCR, one collected in March 2021 scored positive for *L. infantum* (Cq = 37.2), whilst two for *L. tarentolae* (one in June 2020, Cq = 36.2; one in March 2021, Cq = 36.9).

**Table 1:** Variation in antibody titers against *Leishmania infantum* and *Leishmania tarentolae* promastigotes detected by indirect fluorescent antibody (Ab) test according to sampling time (June 2020 and March 2021) and serum dilution (1:80 to 1:640)

Dog ID number	June 2020		March 2021	
	*L. infantum* serum dilution	*L. tarentolae* serum dilution	*L. infantum* serum dilution	*L. tarentolae* serum dilution
1	1:320	1:80	1:1280	1:160
2	1:80	neg	neg	neg
3	neg	neg	1:320	1:160
4	neg	1:160	neg	neg
5	1:160	1:80	1:160	1:80
6	neg	neg	1:640	1:160
7	neg	1:80	1:80	1:80
8	1:80	neg	neg	neg
9	neg	neg	neg	1:160
10	1:80	neg	1:80	neg
11	1:160	1:160	1:160	1:80
12	neg	1:80	neg	neg
13	neg	1:80	1:160	1:160
14	neg	neg	neg	1:160
15	1:640	1:160	1:1280	1:320
16	neg	1:80	neg	neg

*neg* negative

A total of 2306 phlebotomine sand flies (2138 *S. minuta* and 168 *P. perniciosus*) were collected, of which 1281 were females (i.e., 1252 *S. minuta* and 29 *P. perniciosus*). Of female sand flies, 161 scored positive for *Leishmania* spp. (12.6%) by dqPCR (Table 2). Among them, 155 *S. minuta* (95.7%) and two *P. perniciosus* (1.2%) were positive for *L. tarentolae,* whilst four *S. minuta* scored positive for *L. infantum* (2.5%), and only one was co-infected (0.6%) for both *Leishmania* species. In addition, of 22 engorged females tested (14 *S. minuta* and eight *P. perniciosus*), the host mitochondrial *cytb* was amplified from 10 specimens (45.4%, nine *S. minuta* and one *P. perniciosus*). *Cytb* sequences detected in *S. minuta* displayed 99.67% of nucleotide identity with that of *Homo sapiens* (GenBank: JN315800), whilst that from *P. perniciosus* showed 84.4% of identity with lagomorph species *Ochotona cansus* (GenBank: MN547415).

**Table 2:** DNA samples from sand flies tested for *Leishmania infantum* and/or *Leishmania tarentolae* by duplex quantitative PCR

Sand flies	*Leishmania tarentolae*				*Leishmania infantum*				P/T (%)
	P/T (%)	Cq			P/T (%)	Cq			
		M	Min–max	SD		M	Min–max	SD	
*Sergentomyia minuta*	155/1252 (12.4)	29.7	14.7–37.9	6.5	4/1252 (0.3)	35.8	34.7–37.0	0.9	159^a^/1252 (12.7)
*Phlebotomus perniciosus*	2/29 (6.9)	37.05	37.0–37.1	0.06	0/29 (0.0%)	na	na	na	2/29 (6.9)
P/T (%)	157/1281 (12.2)	na	na	na	4/1252 (0.3)	na	na	na	161/1281 (12.6)

The mean (M), minimum (Min), maximum (Max) and standard deviation (SD) values of the cycle of quantification (Cq) are reported

*P/T* Positive/total, *na* not applicable

^a^One *S. minuta* specimen co-infected for *Leishmania* spp.

**Fig. 3:**
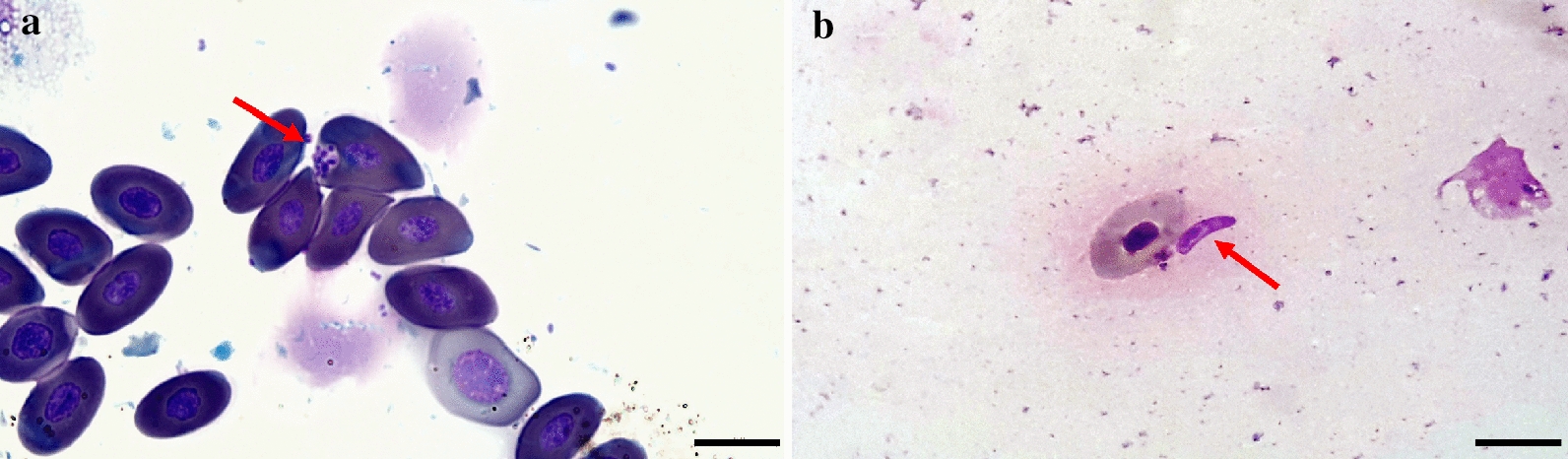
*Leishmania tarentolae* forms in blood from *Podarcis siculus* lizard. **a** Amastigote-like forms associated to erythrocyte (red arrow). **b** Promastigote-like form (red arrow). Scale bar 10 µm

**Fig. 4:**
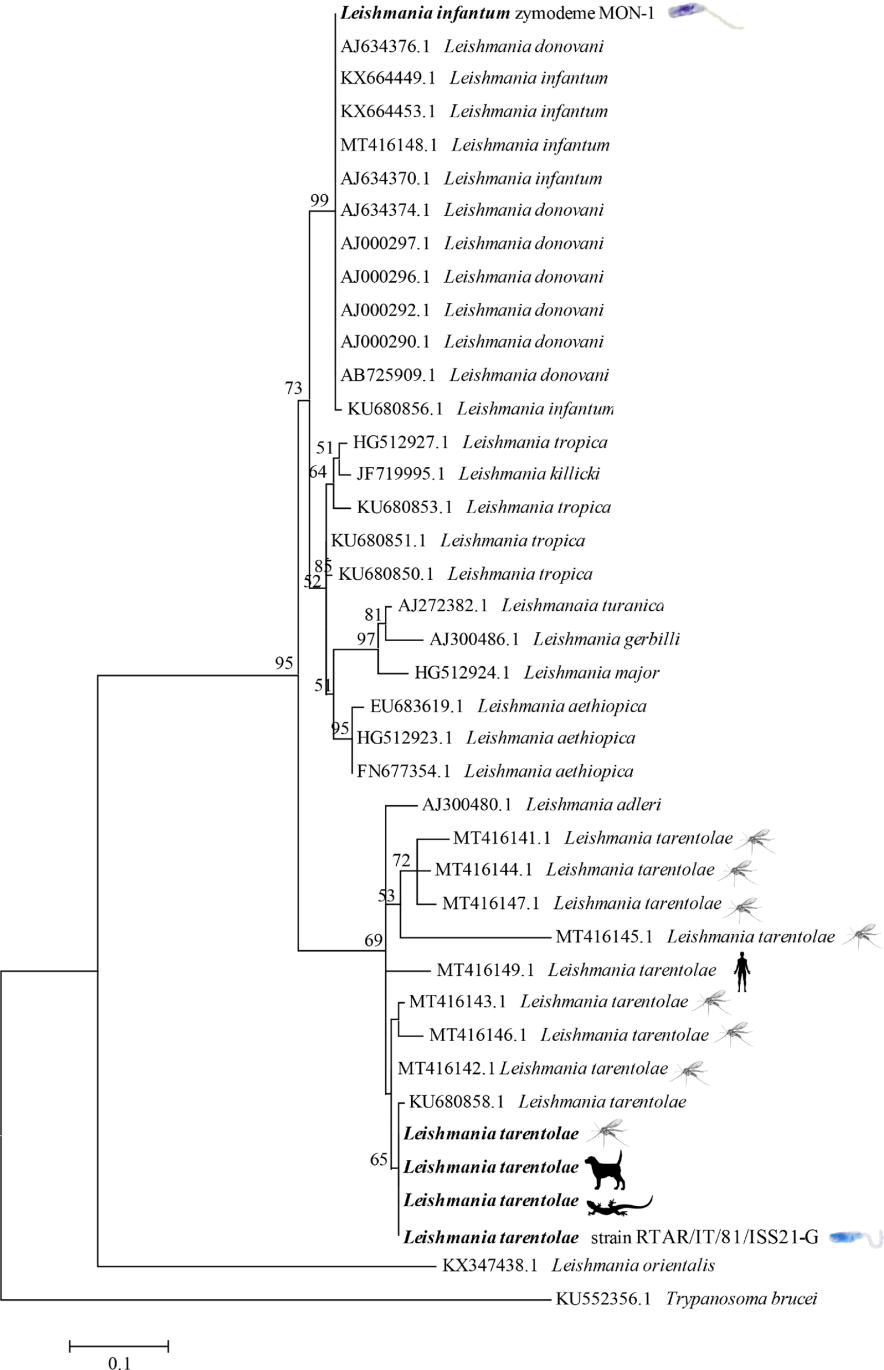
Phylogenetic tree based on *Leishmania* ITS1 sequences inferred using the maximum likelihood method based on the Kimura 2-parameter model. Bootstrap values (> 50%) are shown near the nodes. *Trypanosoma brucei* is used as outgroups. Scale bar indicates nucleotide substitution per site. *Leishmania* spp. sequenced in this study are in bold. Silhouettes represent human, dog, lizard, *Sergentomyia minuta* sand fly and cultured promastigotes of *Leishmania infantum* and *Leishmania tarentolae*

*Podarcis siculus* lizards (*n* = 28) were captured in the same study area (Fig. 1d), including 14 males and 14 females, whereas no snakes or geckoes were collected. Cytological blood smear examination revealed *Leishmania* spp. amastigote-like forms inside erythrocytes (Fig. 3a) and promastigote-like (Fig. 3b) forms in one lizard. Out of 224 lizard tissue samples examined by dqPCR, 22 samples (i.e., intestine, heart, kidneys, liver, lungs, spleen and skeletal muscle) scored positive for *L. tarentolae*, corresponding to 10 positive lizards (35.7%). Lungs had the highest number of positive samples (six, 21.4%), whereas the lowest Cq value (24.7) was recorded from liver (Table 3). Lizard blood, tails and fecal samples were all negative by dqPCR. BLASTn analysis of ITS1 sequences confirmed the *L. tarentolae* species identification showing a nucleotide identity of 98.7% with the reference sequence (GenBank: KU680858) available in the GenBank database and with *L. tarentolae* strain RTAR/IT/81/ISS21-G.6c. The phylogram of ITS1 showed a close phylogenetic relationship by clustering all *L. tarentolae* sequences herein obtained in a species-specific clade (*Sauroleishmania*), with the exclusion of the other *Leishmania* species (bootstrap value of 95%) (Fig. 4). Sequences obtained for *L. tarentolae* from lizards, dogs and sand flies were deposited in GenBank (MW832546, MW832547, MW832548).

**Table 3:** Positivity for *Leishmania tarentolae* in different tissue samples from lizards (*Podarcis siculus*) tested by duplex quantitative PCR

Intestine				Heart				Kidney				Liver				Lung				Spleen				Skeletal muscle			
P/T (%)	Cq			P/T (%)	Cq			P/T (%)	Cq			P/T (%)	Cq			P/T (%)	Cq			P/T (%)	Cq			P/T (%)	Cq		
	M	Min–max	SD		M	Min-max	SD		M	Min–max	SD		M	Min-max	SD		M	Min-max	SD		M	Min–max	SD		M	Min–max	SD
1/28 (3.6)	37.7	na	na	4/28 (14.2)	33.2	29.5–35.2	2.6	2/28 (7.1)	29.8	26.7–33.0	4.5	4/28 (14.2)	32.1	24.7–37.1	5.7	6/28 (21.4)	34.7	32.5–37.5	1.7	4/28 (14.3)	34.0	31.9–36.5	2.2	1/28 (3.6)	33.6	na	na

The mean (M), minimum (Min), maximum (Max) and standard deviation (SD) values of the cycle of quantification (Cq) are reported

*P/T* Positive/total, *na* not applicable

## Discussion

Data herein presented suggests that dogs may be exposed to *L. tarentolae*, a species largely disregarded by the scientific community since it is merely considered a saurian-associated trypanosomatid, yet it occurs in sympatry with *L. infantum*. In addition, dogs after initial exposure against promastigotes of *L. tarentolae* may then seroconvert, remaining seropositive even during the non-transmission sand fly season, suggesting a persistent rather than transient presence of *L. tarentolae* in a non-permissive host. This event may happen in endemic areas where reptiles, herpetophilic sand flies and dogs share the same environment, and both *Leishmania* spp. occur in sympatry.

While *L. tarentolae* has been previously reported exclusively infecting geckoes (i.e., *Tarentola annularis* and *T. mauritanica*) [53–55], the detection in lacertid lizards, *P. siculus*, is a new finding, which could be of major importance to better understand the epidemiology and host preference of this protozoan. The occurrence of *L. tarentolae* in lizards was confirmed both by the detection of *Leishmania* amastigote-like forms in erythrocytes (Fig. 3a) and by cPCR and dqPCR. At cytology, the *Leishmania* forms differ from those of *L. infantum* in that amastigote-like forms infected erythrocytes rather than leucocytes, with possible promastigote-like forms circulating freely in blood (Fig. 3b). The life cycle of *L. tarentolae* is yet to be fully unraveled, though promastigotes and amastigote-like forms have been previously recorded in blood and intestinal lumen from geckoes [10, 22]. Unexpectedly, rather than blood and feces, results of the dqPCR indicated that organs, such as lungs and liver of lizards, are the preferential samples for diagnosing the infection by *L. tarentolae*, probably due to a low parasitic load of promastigotes/amastigotes-like forms in blood. Indeed, parenchymatous organs showed higher amount of *L. tarentolae* DNA, which agrees with higher parasitic loads detected. Blood is not considered the ideal sample for the molecular detection of *Leishmania* spp. due to the low circulation of the parasite [4]. This could represent a hindrance for the molecular identification in mammals, despite the detection of two samples positive with high Cq values.

*Podarcis* lacertid lizards (commonly known as wall lizards) are synanthropic reptiles, which may play a role as reservoirs of other pathogens causing zoonotic diseases such as Lyme disease and rickettsiosis [56, 57]. These reptiles live in wall cracks, under stones and anywhere they find shelter and food, with a wide distribution throughout the Mediterranean basin [58]. The microhabitats where lizards live are similar to that of breeding and resting sites of sand flies [16]. The high prevalence of infection in lizards overlaps the abundance of herpetophilic *S. minuta* and of *P. perniciosus*, which is the main vector of *L. infantum* [59]. Nonetheless, the finding of *S. minuta* as the most abundant species (92.7%) compared to *P. perniciosus* (7.3%) was already observed in other dog shelters from southern Italy where *L. infantum* is prevalent, such as in Apulia [16], Sicily [60–63], as well as Morocco [64], Portugal [65] and Spain [21]. In addition, the low number of *P. perniciosus* collected may be correlated to the species phenology and environmental preferences. Indeed, *P. perniciosus* is more abundant in domestic or peri-urban settings, and *S. minuta* in rural or wild areas, similar to the characteristics of the studied shelter [66]. As for many other species of phlebotomine sand flies, *S. minuta* displays a rather catholic feeding behavior [5] depending on host availability. The detection of human blood in *S. minuta* suggests the opportunistic attitude of this species, as already demonstrated in Sicily where 64% of engorged sand flies scored positive for human blood [63]. The ectoparasiticide treatment of dogs could have affected the sand fly species composition, similarly to a previous study from a dog shelter where a group of animals were treated with a combination of 10% imidacloprid/4.5% flumethrin collar, and the remaining were left untreated [67]. In that study, *S. minuta* was the most common sand fly species identified (66.6%) throughout a collection period of 2 years, followed by *P. perniciosus* (15.1%), *Phlebotomus neglectus* (8.8%) and *Phlebotomus papatasi* (0.23%). Although *S. minuta* has been found molecularly positive for *L. tarentolae*, the vector capacity has never been demonstrated. However, transmission of this *Leishmania* sp. most likely occurs as described for mammalian *Leishmania*, through a pool feeding mechanism [36]. Also, the direct ingestion of the sand fly by lizards cannot be ruled out [36]. Given that the dog population was under an ectoparasiticide treatment and considering the high abundance of *S. minuta*, dogs could have ingested infected *L. tarentolae* sand flies. Another peculiar result of this study is the lack of reptile blood in the engorged *S. minuta* analyzed. This can agree with the hypothesis of a reduced density of preferred reptile hosts in the shelter area, as a consequence of the high predatory pressure exerted by dogs. Hence, further studies are advocated to better elucidate the reptilian and mammalian interactions in the life cycle of *L. tarentolae*.

The molecular detection of *L. tarentolae* in the blood of two dogs is unprecedented, and the exposure to this parasite was confirmed by the seropositivity in 16 dogs, of which eight scored positive for both *Leishmania* species and five against promastigotes of *L. tarentolae* only. This result is new to science, since IFAT using promastigotes of *L. tarentolae* was herein described for the first time. Although the IFAT method reported should be further validated using serum samples of animals purposely infected with both *Leishmania* spp., *L. tarentolae* exposure has been previously demonstrated to be associated with transient infections in mammals [30–33]. Positive dqPCR blood samples for both species of *Leishmania* were from seronegative dogs at both time points, suggesting a recent or transient presence of the parasite for which the animal had not yet seroconverted. In addition, the exposure of animals to this protozoon is also supported by the detection of *L. tarentolae* in two *P. perniciosus*, which usually feed on dogs*.* The association of this *Leishmania* species to sand flies of the genus *Phlebotomus* was already described in 6.6% of *Phlebotomus perfiliewi* examined in Central Italy [14].

Though the seropositivity of dogs against promastigotes of *L. tarentolae* does not imply the reservoir competence of canids, these data are of medical and veterinary relevance. Indeed, the detection of a significant reduction in anti-*L. infantum* antibody titers in 55.4% of *L. infantum*-seropositive and clinically healthy dogs from the same shelter was recently demonstrated after sampling one year apart [68]. A large proportion of these animals (44.4%) became seronegative (i.e., below the cut-off value of 1:80), further suggesting a possible *L. tarentolae* transient exposure. Indeed, although the IFAT is considered the gold standard for the diagnosis of *L. infantum*, as it is based on the visualization of the immunofluorescence on the whole promastigotes, cross-reactions with highly similar species of *Leishmania* may occur. This event was observed in eight dogs which had titers for both species. However, co-infections could also have caused cross-reactivity, given the discrepancies in titers for both species (e.g., dog positive for *L. infantum* with titers 1:1280 and to *L. tarentolae* with 1:160). Given the relevance of serology in epidemiological studies and in the management of diseased patients, the variations in antibody titers requires careful examination. Under the above circumstances, considering that the IFAT for the detection of antibodies against *Leishmania* promastigotes represents the reference serological method for CanL diagnosis and screening, as well as for clinical staging and therapeutic purposes [69, 70], the cross-reactivity between the two species of *Leishmania* might directly impact the interpretation of CanL-related clinical signs, prognosis and treatment. Finally, the sympatric occurrence of *L. infantum* and *L. tarentolae* in sand flies (e.g., co-infection in *S. minuta*) in the specific epidemiological context herein studied could result in hybridization events between these two species*.* This event has been previously experimentally confirmed for *L. infantum* and *Leishmania major* in *Lutzomyia longipalpis* [71]. The possibility of genetic exchange and hybridization events could have implications for the pathogenicity and visceralization capacity of an otherwise innocuous species such as *L. tarentolae*. However, these hypotheses need further research.

## Conclusions

Under specific epidemiological contexts where canids, reptiles, herpetophilic sand flies, *L. infantum* and *L. tarentolae* occur in sympatry, dogs may be exposed to *L. tarentolae*. Results of this study further suggest the low host specificity of *L. tarentolae* in the ability to infect other reptiles (i.e., lacertid lizards) and likely mammals on which *S. minuta* may feed. Serological findings indicate that a cross-reactivity for both species of *Leishmania* may occur, having diagnostic and clinical implications for seropositive healthy dogs. Future studies should focus on determining the prevalence of *L. tarentolae* infection in dogs and its possible interactions with *L. infantum* in areas where they are sympatric.

## Data Availability

All data generated or analyzed during this study are included in this published article. The sequences generated in this study were deposited in GenBank (MW832546, MW832547, MW832548).

## Abbreviations

bp: Base pair
CanL: Canine leishmaniasis
CDC: Centers for Disease Control and Prevention
Cq: Quantitation cycle
cPCR: Conventional PCR
*cytb*: Cytochrome b
dqPCR: Duplex real-time PCR
EDTA: Ethylenediamine tetraacetic acid
IFAT: Immunofluorescence antibody test
IgG: Immunoglobulin G
PCR: Polymerase chain reaction
